# Acuminatol and Other Antioxidative Resveratrol Oligomers from the Stem Bark of *Shorea acuminata*

**DOI:** 10.3390/molecules17089043

**Published:** 2012-07-30

**Authors:** Norhayati Muhammad, Laily B. Din, Idin Sahidin, Siti Farah Hashim, Nazlina Ibrahim, Zuriati Zakaria, Wan A. Yaacob

**Affiliations:** 1 School of Chemical Sciences and Food Technology, Faculty of Science and Technology, Universiti Kebangsaan Malaysia, UKM Bangi 43600, Selangor D.E., Malaysia; Email: norhayatimuhammad@ymail.com (N.M.); lbdin@ukm.my (L.B.D.); 2 Faculty of Mathematics and Natural Sciences, Haluoleo University, Kendari 93232, Sulawesi Tenggara, Indonesia; Email: sahidin02@yahoo.com; 3 School of Biosciences and Biotechnology, Faculty of Science and Technology, Universiti Kebangsaan Malaysia, UKM Bangi 43600, Selangor D.E., Malaysia; Email: farah_samura@yahoo.com (S.F.H.); nazlina@ukm.my (N.I.); 4 Malaysia-Japan International Institute of Technology, Universiti Teknologi Malaysia Kuala Lumpur, Jalan Semarak, Kuala Lumpur 54100, Malaysia; Email: zuriz@ic.utm.my

**Keywords:** Dipterocarpaceae, *Shorea acuminata*, resveratrol oligomer, acuminatol, antioxidant activity, cytotoxicity

## Abstract

A new resveratrol dimer, acuminatol (**1**), was isolated along with five known compounds from the acetone extract of the stem bark of *Shorea acuminata*. Their structures and stereochemistry were determined by spectroscopic methods, which included the extensive use of 2D NMR techniques. All isolated compounds were evaluated for their antioxidant activity using the 2,2-diphenyl-1-picrylhydrazyl (DPPH) radical scavenging activity (RSA) and the β-carotene-linoleic acid (BCLA) assays, and compared with those of the standards of ascorbic acid (AscA) and butylated hydroxytoluene (BHT). All compounds tested exhibited good to moderate antioxidant activity in the DPPH assay (IC_50_s 0.84 to 10.06 mM) and displayed strong inhibition of β-carotene oxidation (IC_50_s 0.10 to 0.22 mM). The isolated compounds were evaluated on the Vero cell line and were found to be non-cytotoxic with LC_50_ values between 161 to 830 µM.

## 1. Introduction

Plants from the Dipterocarpaceae, Gnetaceae and Vitaceae families are known as rich sources of resveratrol oligomers [1]. There have been reports of 275 new resveratrol oligomers from these species between 1995 and 2008 [2]. Many studies have suggested that these groups of constituents exhibited a range of biological activities [3] which included antioxidant [4,5,6,7,8], antimicrobial [9], anti-inflammatory [10], anti-hepatotoxicity [11], anti-tumor [12], cytotoxic effects [6] and other activities [2]. Previous antioxidative studies conducted on oligomers of vitisinols B, C, D; (+)-ε-viniferin, (−)-viniferal, ampelopsin C and (+)-vitisin C from the roots of *Vitis thunbergii* (Vitaceae) have shown that they have strong free RSA with IC_50_ values between 2.8 and 6.6 µM [1]. New resveratrol trimers and tetramer of wilsonols A, B, C and diviniferin B from *V. wilsonae* also exhibited potent antioxidant activities towards DPPH with IC_50_ values of 103.5, 195.4, 182.2 and 175.3 µM respectively [13]. Structure-activity relationship studies revealed that the DPPH RSA of resveratrol dimers isolated from *Cyperus longus* (Cyperaceae) of longusols A, B, C; longusone A and *trans*-scirpusins A, B was stronger (IC_50_s 2.8 to 9.3 µM) than those of the monomers piceatanol and resveratrol (IC_50_s 11 and 24 µM, respectively) [5]. The DPPH RSA study previously conducted on resveratrol oligomers isolated from *Parthenocissus laetevirens* (Vitaceae) of laetevirenols A and B containing an unusual phenanthrene moiety which exhibited much stronger antioxidant activities (38.4 and 37.3 µM) compared to those without that moiety of laetevirenols C-E (110.8, 128.0 and 158.2 µM) [7]. An unusual resveratrol hexamer of chunganenol isolated from *V. chunganensis*, which was composed of more than five monomers, exhibited much stronger DPPH RSA than two resveratrol trimers from the same species of (+)-gnetin H and (+)-amurensin G (with respective values of 37.3; 251.0 and 138.0 µM) [8].

**Figure 1:**
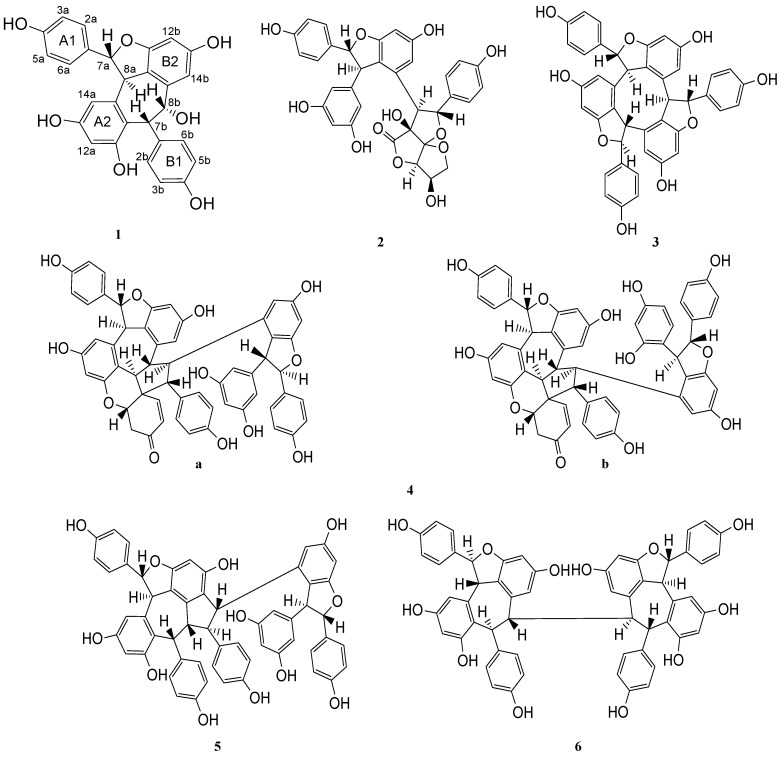
Structures of **1**–**6**.

*Shorea* is the largest genus in the Dipterocarpaceae family. To date, about 26 resveratrol oligomers have been successfully isolated from this genus [1]. *Shorea acuminata* Dyer is a timber tree which is classified as light Red Meranti and locally known as Meranti Rambai Daun. The distribution of the species ranges from the Malay Peninsula to Sumatra and up to the Lingga Archipelago. It occurs on low-lying and well-drained land, but it is more abundant in hilly areas up to 300 m [14]. A previous phytochemical study on *Shorea acuminata* resin had resulted in the characterization of 2α,3α-dihydroxyolean-12-en-28-oic, mangiferonic, 2α-hydroxyursolic and asiatic acids [15]. In our ongoing search for resveratrol oligomers, we now report the isolation and structural elucidation of a new resveratrol oligomer derivative named acuminatol (**1**) and the five known compounds laevifonol (**2**) [16], (+)-α-viniferin (**3**) [17], shoreaketone (**4**) [18], vaticanol B (**5**) [19] and (−)-hopeaphenol (**6**) [20] (Figure 1). This is the first phytochemical report on resveratrol oligomers isolated from *S. acuminata* that incorporates their antioxidant activity against DPPH radical and BCLA, and also their cytotoxic property against Vero cells.

## 2. Results and Discussion

### 2.1. Structure Elucidation

Compound **1** was isolated as a yellow amorphous solid, mp 186–188 °C, 
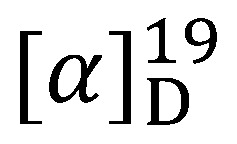
 –42° (c 0.00024, MeOH), which exhibited a molecular ion peak in the negative-ion high resolution ESIMS at [M−H]^–^*m/z* 469.1275 (calcd. 469.1293) attributable to the molecular formula C_28_H_22_O_7_, which corresponded to a resveratrol dimer. This assumption was reinforced by the UV and IR absorption data, together with the ^1^H- and ^13^C-APT NMR data, which was assigned by the interpretation of the HMQC, HMBC, ^1^H-^1^H COSY and NOESY spectra (Table 1). UV absorption λ_max_ nm (MeOH): 282; IR (KBr) ν_max_ cm^–1^: 3366 (OH), 2922 (C–H aliphatic), 1602, 1516 and 1449 (C=C aromatic), 1249 and 1175 (C–O oxyaryl), and 835 (*p*-disubstituted benzene).

**Table 1:** ^13^C- and ^1^H-NMR spectral data * of **1**.

Position	δ_C_	δ_H_ mult. ( *J* in Hz)	^13^C-^1^H HMBC	^1^H-^1^H COSY	^1^H-^1^H NOESY
1a	129.9	-	3a(5a), 8a	-	
2a/6a	129.3	7.09 d (8.6)	7a	3a(5a)	8a, 14a, 7a, 3a(5a)
3a/5a	115.2	6.76 d (8.6)	OH_4a_	2a(6a)	2a(6a), OH_4a_
4a	158.9	-	3a(5a), OH_4a_	-	-
7a	87.5	5.67 d (11.7)	2a(6a), 8a	8a	8a, 14a, 2a(6a)
8a	49.1	4.19 d (11.7)	7a, 14a	7a	7a, 2a(6a), 2b(6b)
9a	141.3	-	7a, 8a, 8b	-	-
10a	120.3	-	7b, 14a, 12a, OH_11a_	-	-
11a	156.5	-	7b, 12a, OH_11a_	-	-
12a	100.5	6.41 d (1.5)	14a, OH_13a_	14a	14a, OH_11a_
13a	157.7	-	OH_13a_	-	-
14a	104.6	6.18 br s	OH_13a_	12a	7a, 12a, 2a(6a), OH_13a_
1b	131.9	-	7b, 3b(5b)	-	-
2b/6b	129.1	7.13 d (8.2)	2b(6b)	3b/5b	7b, 3b(5b), 8a
3b/5b	114.2	6.57 d (8.2)	OH_4b_	2b(6b)	2b(6b)
4b	155.0	-	2b(6b), OH_4b_	-	-
7b	45.0	5.46 br s	-	8b	8b, 2b(6b)
8b	71.0	5.09 br s	7b, 14b, 9a	7b	7b, 14b
9b	141.6	-	7b, 8a	-	-
10b	116.7	-	8a, 12b, 14b	-	-
11b	158.1	-	-	-	-
12b	95.4	6.11 d (1.8)	14b, OH_13b_	14b	14b, OH_13b_
13b	156.0	-	-	-	-
14b	106.4	6.94 d (1.8)	OH_13b_	12b	8b, 12b, OH_13b_
OH_8b_	-	3.63		-	
OH_11a_	-	8.57		-	
OH_4a_	-	8.54		-	
OH_13b_	-	8.23		-	
OH_13a_	-	8.22		-	
OH_4b_	-	8.05		-	

* ^13^C- and ^1^H-NMR spectra were obtained at 100 and 400 MHz (acetone-*d_6_*), respectively.

The ^1^H-NMR spectral data of **1** exhibited the presence of two sets of *ortho-*coupled aromatic protons which can be assigned to two 4-hydroxyphenyl groups [ring A1: δ 7.09 (2H, d, *J* = 8.6 Hz, H-2a and 6a), δ 6.76 (2H, d, *J* = 8.6 Hz, H-3a and 5a), and ring B1: δ 7.13 (2H, d, *J* = 8.2 Hz, H-2b and 6b), δ 6.57 (2H, d, *J* = 8.2 Hz, H-3b and 5b)], two sets of *meta*-coupled aromatic protons on two 1,2,3,5-tetrasubstituted benzene rings [ring A2: δ 6.18 (1H, br s, H-14a), δ 6.41 (1H, d, *J* = 1.5 Hz, H-12a), and ring B2: δ 6.94 (1H, d, *J* = 1.8 Hz, H-14b), δ 6.11 (1H, d, *J* = 1.8 Hz, H-12b)], five phenolic hydroxyl groups (δ 8.05, 8.22, 8.23, 8.54 and 8.57), and one aliphatic hydroxyl group (δ 3.63). The ^1^H-NMR spectrum also showed two pairs of aliphatic methine protons coupled successively: H-7a (δ 5.67, d, *J* = 11.7) and H-8a (δ 4.19, d, *J* = 11.7); H-7b (δ 5.46, br s) and H-8b (δ 5.09, br s) as shown in Table 2. The large coupling constant of H-7a and H-8a (11.7 Hz) in compound **1** indicated that the protons were in *trans* orientation [21], which is also exhibited by its stereoisomers, namely (+)-ampelopsin A (11.7 Hz) [22], (−)-hemsleyanol A (9.8 Hz) [17] and (+)-balanocarpol (9.3 Hz) [23]. On the other hand, H-7b and H-8b of **1** gave two broad singlets as those in (+)-balanocarpol [23], which proved that the two protons were in *cis* orientation. However, *trans*–H-7b~H-8b in (+)-ampelopsin A [22] and (−)-hemsleyanol A [17] gave coupling constants of 5.0 and 5.9 Hz for H-7b whereas H-8b produced broad singlet and broad doublet respectively.

**Table 2:** Chemical shifts of aliphatic proton pairs of H-7a~H-8a and H-7b~H-8b for stereoisomers **1**, (+)-ampelopsin A [22], (−)-hemsleyanol A [17] and (+)-balanocarpol [23].

Compound	δ_H_ mult. ( *J* in Hz)			
	7a	8a	7b	8b
1 ^a^	β: 5.67 (d, 11.7)	α: 4.19 (d, 11.7)	β: 5.46 (br s)	β: 5.09 (br s)
(+)-Ampelopsin A ^b^	α: 5.77 (d, 11.7)	β: 4.17 (br d, 11.7)	α: 5.45 (d, 5.0)	β: 5.42 (br s)
(−)-Hemsleyanol A ^a^	β: 5.75 (d, 9.8)	α: 5.41 (d, 9.8)	α: 5.07 (d, 5.9)	β: 4.76 (br d)
(+)-Balanocarpol ^a^	β: 5.69 (d, 9.3)	α: 5.16 (br d, 9.3)	α: 4.90 (br s)	α: 5.40 (br s)

^a^ Measured in acetone-*d_6_* (400 MHz); ^b^ measured in acetone-*d_6_* (500 MHz).

**Figure 2:**
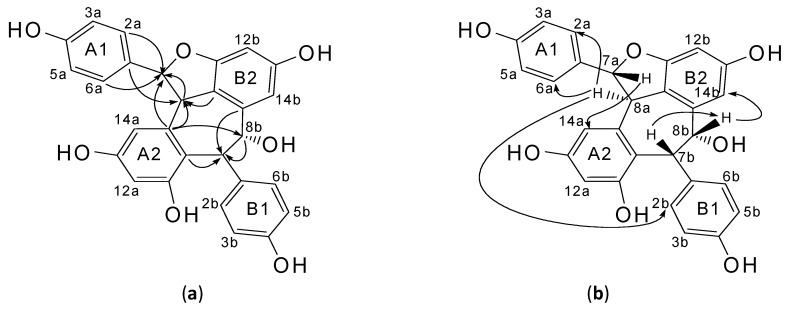
Key HMBC (**a**) and NOE (**b**) correlations for **1**.

Relative configuration of methine protons at C-8a, C-7b and C-8b on a cycloheptane ring in compound **1** can be further verified by the results of NOESY experiment. The absence of NOEs between H-8a and either H-7b or H-8b revealed that H-8a and H-7b~H-8b were in opposite sides of the cycloheptane ring. This also confirmed that both H-7b and H-8b were in the *cis* position. (+)-Balanocarpol where all three hydrogens were on the same side of the cycloheptane ring had significant NOEs between H-8a/H-7b and H-8a/H-8b [23]. Since H-7a and H-8a of **1** were *trans* based on the above ^1^H-NMR spectral data, the relative configuration of methine protons at C-7a, C-8a, C-7b and C-8b were β, α, β and β. Other significant NOEs in support of these observations were between β H-7a/H-14a, β H-7b/β H-8b and β H-8b/H-14b. In the HMBC spectrum (Table 1, Figure 2a), significant correlations were observed between C-2a(6a)/H-7a, C-8a/H-7a, C-9a/H-7a, C-9a/H-8a and C-10b/H-8a, indicated that a pair of benzylic methine protons of β H-7a and α H-8a was assigned to the protons on a dihydrofuran ring. 

There were significant correlations observed between C-9a/H-8b, C-9b/H-7b, C-1b/H-7b, C-11a/H-7b, C-10a/H-7b and C-8b/H-7b. These correlations proved that there was one more pair of methine protons attached to C-7b (β H-7b) and C-8b (β H-8b). The relative stereo structure for compound **1** was confirmed as shown in Figure 1. It should be noted that the above three resveratrol dimers, which were stereoisomers to compound **1**, were previously isolated from *Ampelopsis brevipedunculata* var. *hancei* (Vitaceae) [(+)-ampelopsin A] [22], *H. parvifolia* (Dipterocarpaceae) [(+)-balanocarpol] [23] and *Shorea hemsleyana* (Dipterocarpaceae) [(−)-hemsleyanol A] [17].

**Figure 3:**
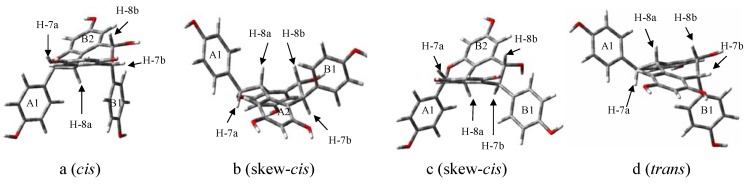
Energy-optimized stereo structures of **1** (**a**), (+)-ampelopsin A (**b**), (−)-hemsleyanol A (**c**) and (+)-balanocarpol (**d**).

All of the four stereoisomers shared a same basic planar structure. However, the chemical shifts for aliphatic protons of H-7a, H-8a, H-7b and H-8b of the four stereoisomers were markedly different as shown in Table 2. These differences have been suggested to be mainly due to the A1 and B1 rings in their energy-optimized conformations as in Figure 3. 

The NOE interactions between H-8a/H-2a(6a), H-7a/H-14a, H-8b/H-14b and H-8a/H-2b(6b) in compound **1** (Figure 3a) and (+)-ampelopsin A (Figure 3b) [22] lead to *cis* and skew-*cis* conformations, while the absence of NOE interaction between H-8a/H-2b(6b) in (+)-balanocarpol (Figure 3d) [23] would suggest that it was in a *trans* conformation. The absence of NOE interaction between H-8a/H-2b(6b) in the skew-*cis* conformation of (−)-hemsleyanol A (Figure 3c) [17], unlike that in the skew-*cis* conformation of (+)-ampelopsin A, was due to the relative positions of H-8a and B1 ring which were farther apart in the actual energy-optimized 3D-model of the molecule. It should be noted that the NOE correlations between H-2a(6a)/H-14a and H-7a/H-8a in compound **1** seem to suggest that the H-7a and H-8a were *cis* to each other with H-7a in α position. However, this contradicted with the large coupling constants between H-7a and H-8a of the *trans* in compound **1** (11.7 Hz) as well as in its stereoisomers of (+)-ampelopsin A (11.7 Hz) [22], (−)-hemsleyanol A (9.8 Hz) [17] and (+)-balanocarpol (9.3 Hz) [23]. The chemical shift for H-8a in compound **1** was more upfield (δ 4.19) like the one found in (+)-ampelopsin A (δ 4.17) [22] when compared to that in (−)-hemsleyanol A (δ 5.41) [17] and (+)-balanocarpol (δ 5.16) [23]. This strongly suggested that the A1 and B1 rings in compound **1** and (+)-ampelopsin A were in *cis* configuration. The chemical shifts for H-8a in (−)-hemsleyanol A and (+)-balanocarpol which appeared 1.23 and 0.98 ppm more downfield indicated that the A1 and B1 rings for the latter were in *trans* configuration (Table 2). Although the A1 and B1 rings for (−)-hemsleyanol A were in *cis* configuration, its B1 ring did not give any deshielding effect like in (+)-balanocarpol since those rings were in the same orientation as shown in Figure 3c,d. The H-8a in (−)-hemsleyanol A was more deshielded than that in (+)-balanocarpol because the former lay within the deshielding area up front to the A1 ring whereas this ring in the latter was a little tilted. Moreover NOE correlations [Table 1, Figure 2b] between H-8a/H-2a(6a) and H-8a/H-2b(6b) in compound **1** positively suggested that its A1, H-8a and B1 were all on the same side of the plane. The results from the energy-optimized conformations as calculated by GaussView 5.0 and Gaussian 09W using DFT-6-31G-(d,p) method, have confirmed the above deductions (Figure 3). Therefore, **1** is a new stereoisomer of these three compounds. 

### 2.2. Antioxidant Activity

#### 2.2.1. DPPH Assay

As shown in Table 3, the scavenging activity of compounds **1** to **6** towards DPPH free radicals was expressed in terms of IC_50_ values. Since lower IC_50_ values indicated stronger ability of the compounds to act as DPPH radical scavengers, it was obvious that the positive controls were excellent DPPH radical scavengers with AscA and BHT exhibited average 6- and 4-fold higher scavenging activities (IC_50_ = 0.68 and 0.95 mM, respectively) compared to compounds **1** to **6** (IC_50_s = 0.84 to 10.06 mM). Compound **5** exhibited excellent RSA with IC_50_ having no significant difference (*p* > 0.05) compared to positive controls of AscA and BHT. The antioxidant activitiy of all compounds and positive controls was in the following order (Table 3): AscA ~ **5** ~ BHT > **4** > **6** > **2** > **3** > **1**. 

**Table 3:** Antioxidant and cytotoxic activities of compounds **1** to **6**.

Compound	DPPH radical scavenging activities (IC_50_, mM)	BCLA method(IC_50_, mM)	Cytotoxic activities on Vero cell lines (LC_50_, µM)
1	10.06 ± 0.05 ^f^	0.18 ± 0.01 ^a^	400
2	4.21 ± 0.23 ^d^	0.22 ± 0.02 ^a^	597
3	6.29 ± 0.05 ^e^	0.18 ± 0.00 ^a^	208
4	1.54 ± 0.10 ^b^	0.11 ± 0.00 ^a^	830
5	0.84 ± 0.02 ^a^	0.10 ± 0.01 ^a^	759
6	2.78 ± 0.16 ^c^	0.10 ± 0.01 ^e^	161
AscA ^z^	0.68 ± 0.00 ^a^	25.19 ± 1.74 ^b^	-
BHT ^z^	0.95 ± 0.05 ^a^	0.09 ± 0.00 ^a^	-

^a-g^ Mean within each column with different letters differ significantly (*p* < 0.05). Each value is presented as mean ± SD (*n* = 3). ^z^ Positive reference standards; IC_50_, 50% inhibition concentration.

These results, especially for the compounds **5**, **4**, **3** and **1**, were in agreement with the recent study on antioxidant activity which revealed that the RSA of the resveratrol oligomers was related to their structures. The multiple phenolic hydroxyl groups of 10, 8, 6 and 5 for the compounds **5**, **4**, **3** and **1** with four, three, three and two *para*-hydroxy groups, respectively, contributed to their being better hydrogen donors. Furthermore, the presence of the extensive double bond conjugation within the compounds which was responsible for electron delocalization has made them good as radical targets [3]. Compounds **6** and **2** with 10 and five aromatic-OHs, and four and two *para*-OHs of did not fit very well into the order. Other related studies by He *et al*. in 2009 [8] on eight stilbene oligomers from *Vitis chunganensis* (Vitaceae), that is, hexamer of chunganenol, tetramers of vitisin A and hopeaphenol (**6**), four trimers and one monomer revealed that chunganenol was the most active, as expected, followed by vitisin A, with hopeaphenol ranking fifth in the order. 

#### 2.2.2. BCLA Assay

The antioxidant activity of the compounds **1** to **6** as well as the positive controls BHT and AscA, as measured by the bleaching of *β*-carotene, are presented in Table 3. It was noted that in this assay, AscA exhibited low antioxidant activity (IC_50_ 25.19 mM) compared to BHT (IC_50_ 0.09 mM) and compounds **1** to **6** (IC_50_ 0.10 to 0.22 mM). These results suggest that AscA is a weak antioxidant despite the fact that it is a well-known, polar antioxidant. Our results are in agreement with the previous report which pointed out that AscA did not show its antioxidant activity under similar assay [24]. In this assay, all compounds exhibited antioxidant activity with no significant difference (*p* > 0.05) than the positive control BHT. These results agree with previous report [1], which indicated that most stilbenoids possess antioxidant activity because they had polyphenol functions in the molecules. 

### 2.3. Cytotoxicity Assay

The results of cytotoxicity evaluation of compounds **1** to **6** as LC_50_ (mM) are shown in Table 3. The compounds were considered safe when their LC_50_s are higher than 100 μM [25]. All compounds tested possessed LC_50_ values more than 100 μM (161 to 830 μM). 

## 3. Experimental

### 3.1. General

IR spectra were recorded on a Perkin Elmer GX FT-IR spectrophotometer (Waltham, MA, USA). UV spectra were measured on Shimadzu UV-160 (200–400 nm, Kyoto, Japan). ^1^H and ^13^C-APT NMR spectra were recorded in acetone-*d_6_* using JEOL ECP400 spectrometer (400 and 100 MHz for ^1^H and ^13^C; Akishima, Japan). Mass spectra were measured in electron spray ionization mode on Bruker (micro TOF-Q; Bremen, Germany) LC-MS spectrometer (ESI-MS in negative mode, Dionex, Sunnyvale, CA, USA). Melting points were determined by Stuart SMP10 melting point apparatus (Burlington, VT, USA) and were uncorrected. Optical rotations were recorded on Jasco Polarimeter P-1020 (Easton, MD, USA) in MeOH. Vacuum liquid chromatography (VLC) was carried out on Si-gel 60 GF_254_ (Merck, Damstadt, Germany), radial chromatography (RC) was done on Si-gel 60 PF_254_ (Merck) and TLC was performed on pre-coated silica gel (Merck, Kieselgel 60 F_254_ 0.25 mm), and detected by UV light (254 nm) or by CeSO_4_ spraying reagent followed by heating. All solvents used were of analytical grades. Absorbance values for BCLA and cytotoxic assays were measured on microplate reader (Labsystem Multiskan Multisoft, Basingstoke, UK) and DPPH assay on Shimadzu UVmini-1240 spectrophotometer (Kyoto, Japan). Dulbecco’s Modified Eagle’s Medium (DMEM) and fetal bovine serum (FBS) used to maintain and supplement the cell, respectively, were from Flowlab (North Ryde, Australia). Samples emulsifying was done on Branson 5200 (Los Angeles, CA, USA) sonicator.

### 3.2. Plant Material

The stem bark of *Shorea acuminata* was collected from Universiti Kebangsaan Malaysia (UKM) Forest Reserve, Bangi, in September 2009. A voucher specimen (UKMB 23520) has been deposited in the UKM Herbarium, and identified by Mr. Sani Miran.

### 3.3. Extraction and Isolation

The dried powder of stem bark of *Shorea acuminata* (1 kg) was macerated with acetone (3 × 5 L, 3 days each) at room temperature. The extract was concentrated using rotary evaporator to yield a brownish acetone extract (49.6 g, 4.96%) that was fractionated by VLC eluted with mixtures of *n*-hexane-EtOAc of increasing polarity. The eluates that showed similar profile on TLC chromatogram were combined to give five fractions A–E. Fraction C (1.5 g) was subjected to RC by eluting with CHCl_3_-MeOH (9:1) to afford compound **3** (4.9 mg). Purification of fraction D (400 mg) by RC using CHCl_3_-MeOH (8.4:1.6) followed by preparative TLC (CHCl_3_-MeOH, 8.2:1.8) gave compounds **1** (12.0 mg), **2** (4.0 mg) and **4** (7.6 mg). Purification of fraction E (600 mg) by RC using CHCl_3_-MeOH (8:2) followed by preparative TLC afforded compounds **5** (22.5 mg) and **6** (5.2 mg).

*Compound* **1**: Yellow amorphous solid, m.p. 186–188 °C, 
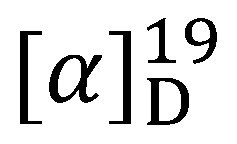
 –42° (c 0.00024, MeOH), UV (MeOH) λ_max_ 282 nm; IR (KBr) ν_max_ cm^–1^: 3366, 2922, 1602, 1516 and 1449, 1249 and 1175, 835. ESIMS (neg): *m/z* 469.1275 [M−H]^–^, calcd: 469.1293; ^1^H- and ^13^C-NMR data, see Table 1.

### 3.4. Antioxidant Assays

#### 3.4.1. DPPH Assay

The antioxidant activity of all six compounds was determined by the DPPH radical scavenging method according to the previous procedure [26] with modification. Each compound was diluted two-fold in a series of five starting from 5.00 mg/mL. The solution of DPPH in methanol (6 × 10^−5^ M) was prepared daily before UV measurements. A 3 mL aliquot of this solution was mixed with 77 μL of the compound solution in a 1-cm path length microcuvette. The mixture was shaken vigorously. The mixtures were then kept in the dark for 15 min at room temperature and the decrease in absorption was measured immediately in a UV-Vis spectrophotometer at 515 nm. The blank solution containing the same amount of methanol and DPPH solution was prepared and its absorption was measured daily. The experiment was carried out in triplicate. AscA and BHT were used as positive controls. The RSA of samples, expressed as percentage inhibition of DPPH, was calculated according to the formula: Inhibition percentage (Ip) = [(A_B_ − A_A_)/A_B_] × 100, where A_B_ and A_A_ are the absorbance values—checked after 15 min—of the blank sample and of the tested sample solutions, respectively. The IC_50_ values, which represented the concentrations of the tested samples and standards that caused 50% RSA of DPPH, were calculated from the plots of inhibition percentages against concentrations.

#### 3.4.2. BCLA Assay

This test was carried out according to the reported method of literature [27] with modification. Approximately 4 mL of a solution of β-carotene in chloroform (1 mg/mL) were pipetted into a flask containing 40 mg of linoleic acid and 400 mg of Tween-40. The chloroform was removed using a rotary evaporator at 40 °C for 5 min, and to the residue, 100 mL of distilled water were added, slowly with vigorous agitation, to form an emulsion. A 96-well micro-titer plate was added with 50 μL of the Part 2.4.1 solution of the test compound and 200 μL of the emulsion, and the absorbance was measured at 450 nm, immediately, against a blank consisting of the emulsion without β-carotene. The plate was allowed to stand at room temperature (20–23 °C), and the absorbance measurements were conducted again at 30 min intervals up to 120 min. All tests were carried out in triplicate. Stable antioxidants of AscA and BHT were used as positive controls. The antioxidant activity (AA) of the test samples was evaluated in terms of bleaching of β-carotene using the formula; AA = [1 − (A_0_ − A_t_)/(A_0_' − A_t_')] × 100, where A_0_ and A_0_' are the absorbance values measured at zero time of the sample and the blank, respectively, and A_t_ and A_t_' are the absorbance measured in the test sample and the blank, respectively, at times up to 120 min. The IC_50_ values, which represented the concentrations of the tested samples and standards that caused 50% bleaching of BCLA, were calculated from the plots of inhibition percentages against concentrations.

### 3.5. Cytotoxicity Assay

The isolated compounds were tested for *in-vitro* cytotoxicity using Vero cells by 3-(4,5-dimethylthiazol-2-yl)-2,5-diphenyltetrazolium bromide (MTT) assay [28]. The Vero cell line was initiated from kidney of a normal adult African green monkey, *Cercopitheus aethiops*, obtained from Virology Laboratory, School of Biosciences and Biotechnology, Faculty of Science and Technology, UKM. Vero cells were maintained in DMEM, supplemented with 10% FBS and cultured at 37 °C in a humidified 5% CO_2_ incubator. The concentration of stock compound solution was 1.0 mg/mL prepared by dissolving 1 mg compound in 50 μL methanol and 950 μL 5% FBS-DMEM. Sonicator was used to emulsify the compounds for 40 min before the two-fold dilutions made in 5% FBS-DMEM to produce a compound solution at concentrations of 0.5, 0.25, 0125 and 0.0625 mg/mL. Briefly, a total of 50 mL of cell suspension with different concentrations were added to each well in the 96-well micro-titer plates. As a positive control, each well from first to third wells was added with cells of 2.5 × 10^5^, 1.25 × 10^5^ and 0.625 × 10^5^ cells/mL. For the negative control (100% cell death, LC_100_), 50 mL of DMEM without cells were added to the twelfth well. Cells of 2.5 × 10^5^ cells/mL were added into the fourth to eleventh wells. The plates were incubated for 48 h at 37 °C with 5% CO_2_ until a monolayer is formed. A total of 50 μL of test compound solution were placed in each well containing the monolayer cells and 50 μL of phosphate buffered saline was added to each control cell well and negative control well (LC_100_), and incubated for 24 h at 37 °C with 5% CO_2_. After the incubation period, MTT (20 μL, 5 mg/mL) was added into each well and the cells incubated for 2 to 4 h, until a purple precipitate was clearly visible under a microscope, the medium together with MTT (190 μL) was aspirated off from the wells, DMSO (100 μL) was added and the plate shaken for 5 min. The absorbance for each well was measured at 540 nm in a micro-titer plate reader and percentage of cell viability (CV) was calculated manually using the formula: CV = [(Abs_sample_ − Abs_negative control_)/(Abs_cell_ − Abs_negative control_)] × 100. A dose-response curve was plotted to enable the calculation of the concentration that kills 50% of the Vero cells (LC_50_).

### 3.6. Statistical Analysis

Values expressed are means of the three replicate determinations ± standard deviation. All statistical analyses were carried out using SPSS 16.00 for Windows. To determine whether there were any differences between activities of samples, variance analysis (one-way ANOVA) was applied to the results. Values of *p* < 0.05 were considered as significant different (α = 0.05).

## 4. Conclusions

The compounds **1** to **6** were found to be potent antioxidants, comparable in activity to the widely used synthetic antioxidant BHT in both assays. The activity was found to mostly increase with the number of phenolic units in the oligomer molecules. The non-toxic nature of all compounds **1** to **6** against normal (Vero) cells should merit further investigation to assess the effectiveness of these compounds in other biological activities including against other cell lines. 

## Supplementary Materials

Supplementary materials can be accessed at: http://www.mdpi.com/1420-3049/17/8//9043/s1.
