# Supplementary material for: Acuminatol and Other Antioxidative Resveratrol Oligomers from the Stem Bark of Shorea acuminata
Source: Molecules. 2012 Jul 30;17(8):9043–55. doi: 10.3390/molecules17089043 (PMC6268933; doi:10.3390/molecules17089043)
Supplement: Supplementary file 1 [file molecules-17-09043-s001.pdf]

Supplementary Materials

Acuminatol and Other Antioxidative Resveratrol Oligomers from the Stem Bark of *Shorea acuminata*

Norhayati Muhammad [1], Laily B. Din [1], Idin Sahidin [2], Siti Farah Hashim [3], Nazlina Ibrahim [3], Zuriati Zakaria [4] and Wan A. Yaacob [1,*]

[1] School of Chemical Sciences and Food Technology, Faculty of Science and Technology, Universiti Kebangsaan Malaysia, UKM Bangi 43600, Selangor D.E., Malaysia; E-Mails: norhayatimuhammad@ymail.com (N.M.); lbdin@ukm.my (L.B.D.)

[2] Faculty of Mathematics and Natural Sciences, Haluoleo University, Kendari 93232, Sulawesi Tenggara, Indonesia; E-Mail: sahidin02@yahoo.com

[3] School of Biosciences and Biotechnology, Faculty of Science and Technology, Universiti Kebangsaan Malaysia, UKM Bangi 43600, Selangor D.E., Malaysia; E-Mails: farah_samura@yahoo.com (S.F.H.); nazlina@ukm.my (N.I.)

[4] Malaysia-Japan International Institute of Technology, Universiti Teknologi Malaysia Kuala Lumpur, Jalan Semarak, Kuala Lumpur 54100, Malaysia; E-Mail: zuriz@ic.utm.my

* Author to whom correspondence should be addressed; E-Mail: wanyaa@ukm.my; Tel.: +603-8921-5424; Fax: +603-8921-5410.

Acuminatol and Other Antioxidative Resveratrol Oligomers from the Stem Bark of *Shorea acuminata*.

Figure 1. ^{1}H-NMR spectrum of compound **1**.

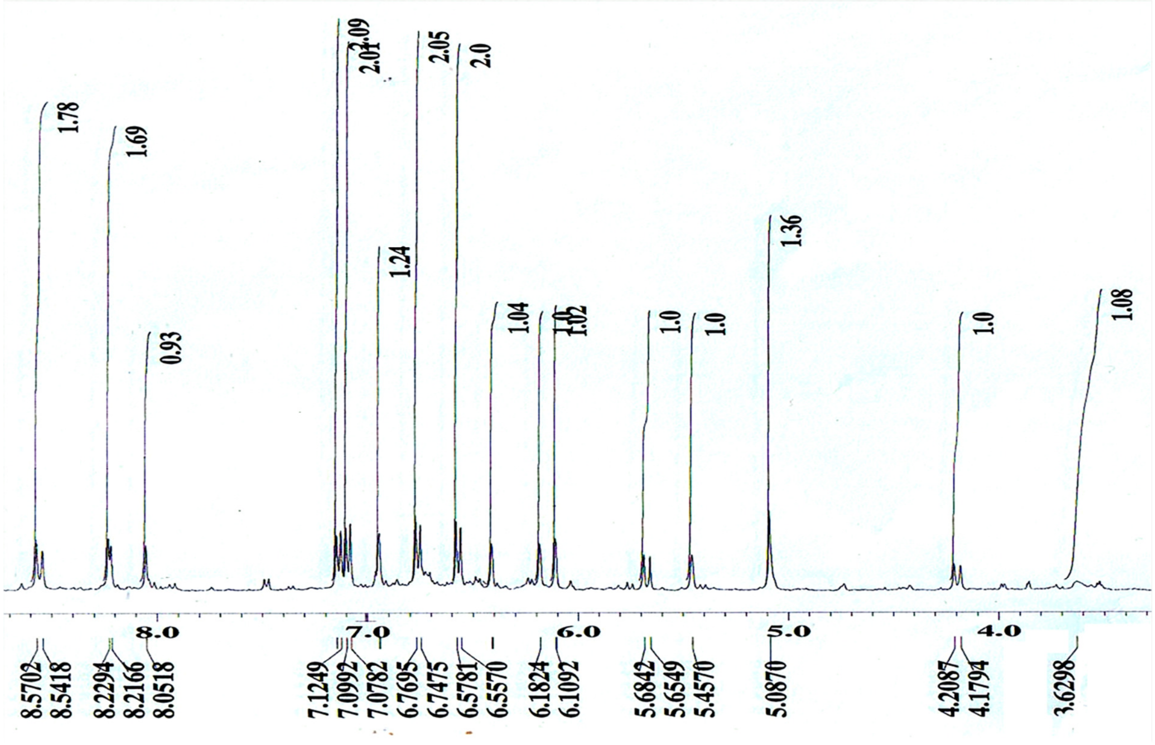

Figure 2. ^{13}C-APT NMR spectrum of compound **1**.

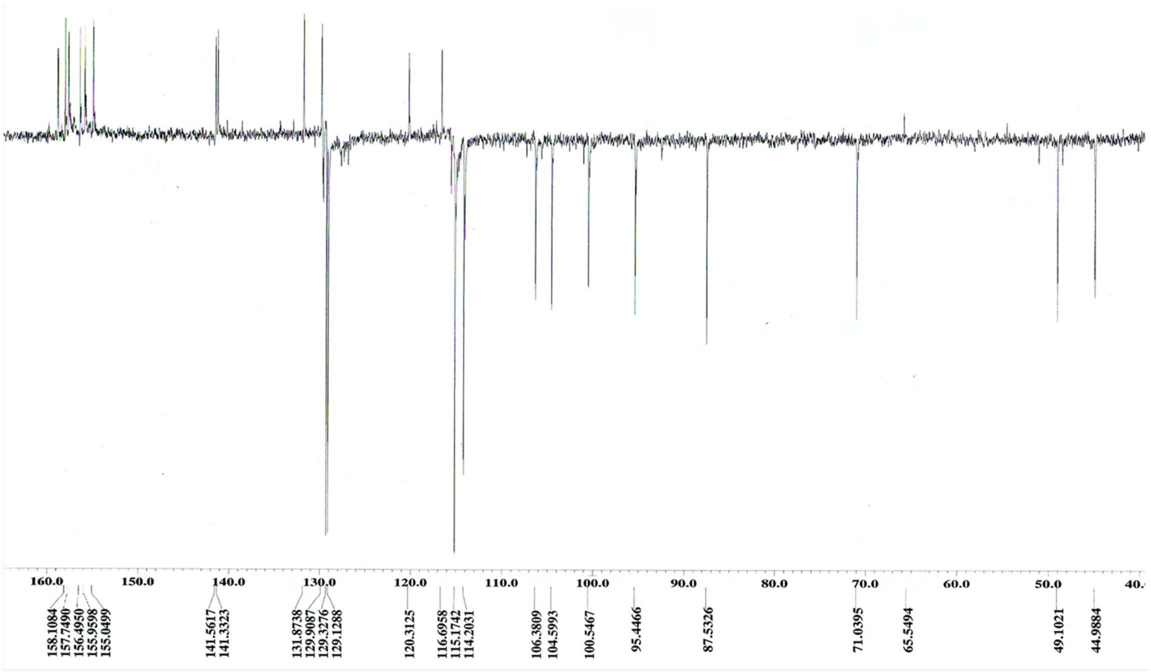

Figure 3. HMQC spectrum of compound **1**.

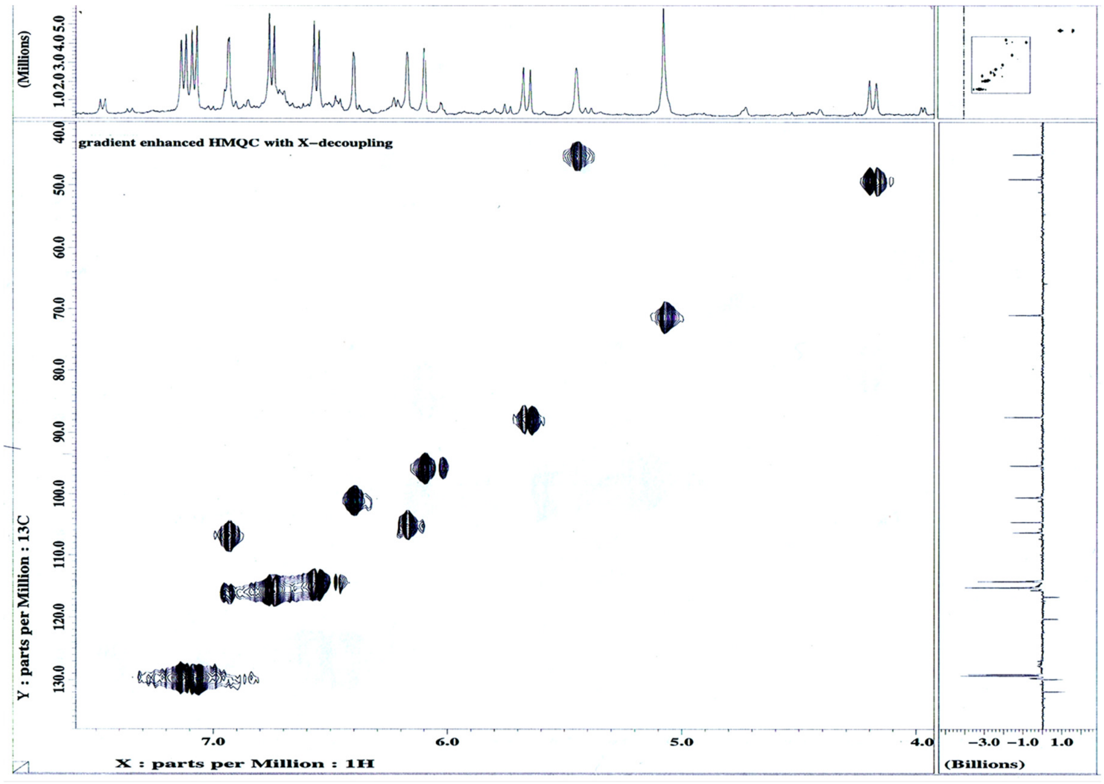

Figure 4. COSY spectrum of compound **1**.

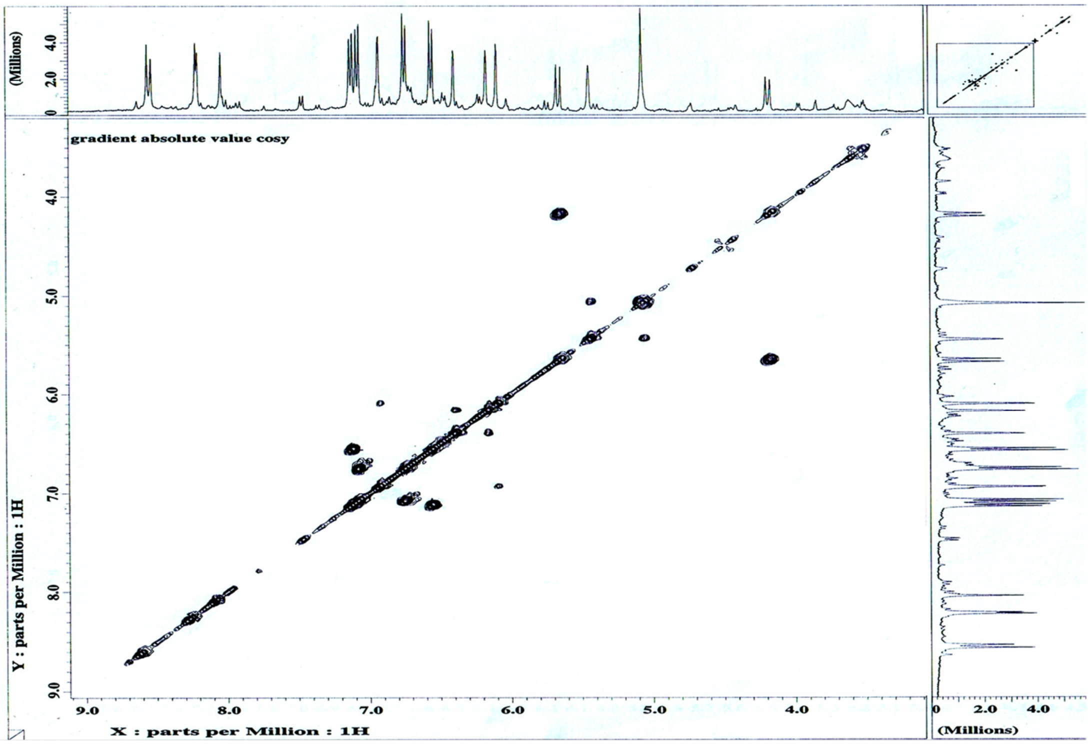

Figure 5. NOESY spectrum of compound **1**.

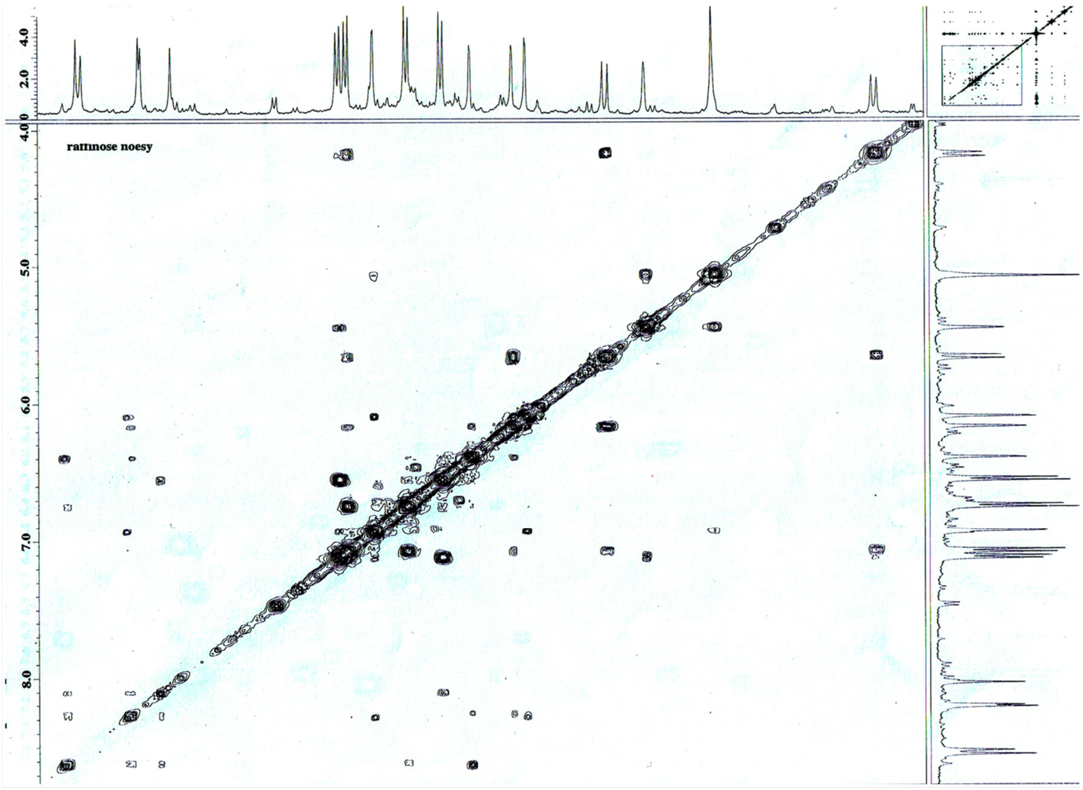

Figure 6. HMBC spectrum of compound **1**.

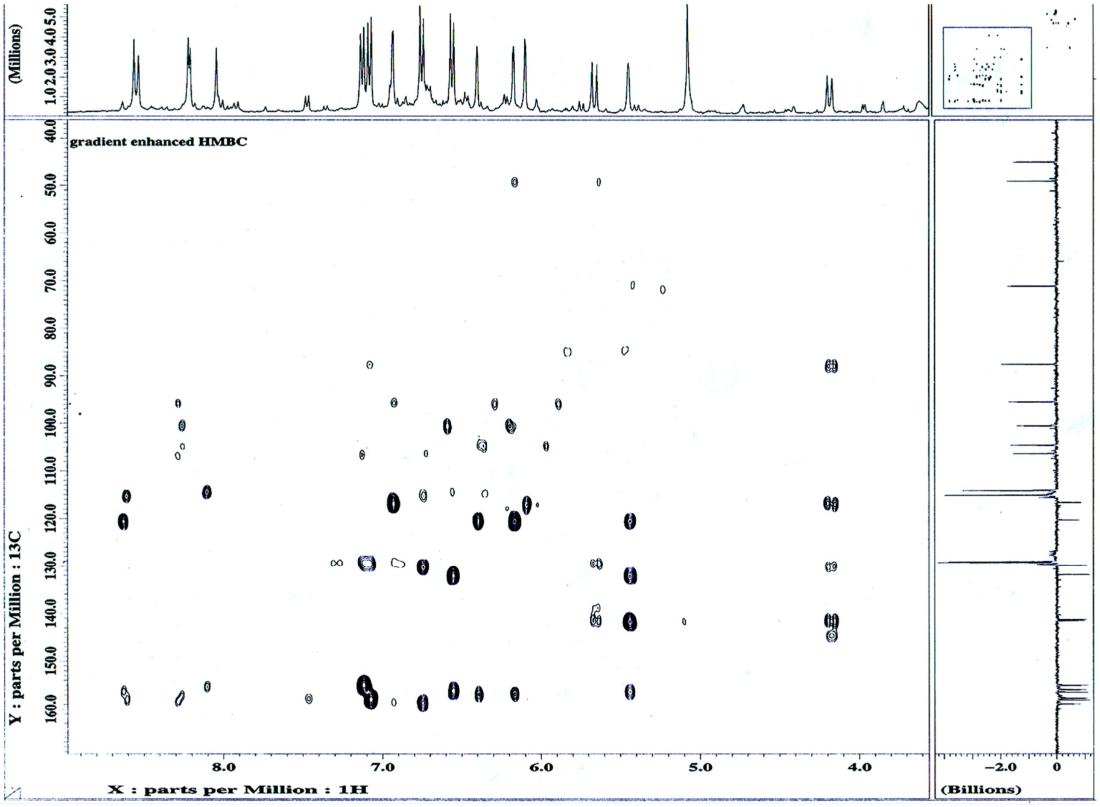

Figure 7. Mass spectrum of compound **1**.

Mass Spectrum Molecular Formula Report

Analysis Info

Analysis Name	D:\Data\sample 2011\norhayati\101121.d	Acquisition Date	11/10/2011 11:34:13 AM
Method	direct infuse -ve.m	Operator	Alefee
Sample Name	SAK 151742462	Instrument / Ser#	micrOTOF-Q 86
Comment	10.11.2011		

Acquisition Parameter

Source Type	ESI	Ion Polarity	Negative	Set Nebulizer	1.0 Bar
Focus	Not active	Set Capillary	3500 V	Set Dry Heater	190 °C
Scan Begin	50 m/z	Set End Plate Offset	-500 V	Set Dry Gas	5.0 l/min
Scan End	1100 m/z	Set Collision Cell RF	450.0 Vpp	Set Divert Valve	Waste

Generate Molecular Formula Parameter

Formula, min.	C20				
Formula, max.	N0				
Measured m/z	469.128	Tolerance	10 mDa	Charge	-1
Check Valence	no	Minimum	0	Maximum	0
Nirogen Rule	no	Electron Configuration	both		
Filter H/C Ratio	no	Minimum	0	Maximum	3
Estimate Carbon	yes				

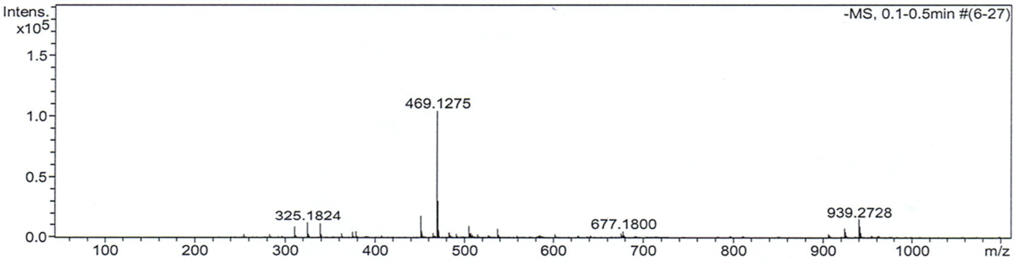

Sum Formula	Sigma	m/z	Err [ppm]	Mean Err [ppm]	Err [mDa]	rdb	N Rule	e⁻
C 21 H 25 O 12	0.033	469.1351	16.24	15.99	7.62	9.50	ok	even
C 28 H 21 O 7	0.008	469.1293	3.72	3.46	1.74	18.50	ok	even
C 35 H 17 O 2	0.046	469.1234	-8.80	-8.94	-4.13	27.50	ok	even

Figure 8. UV spectrum of compound **1**.

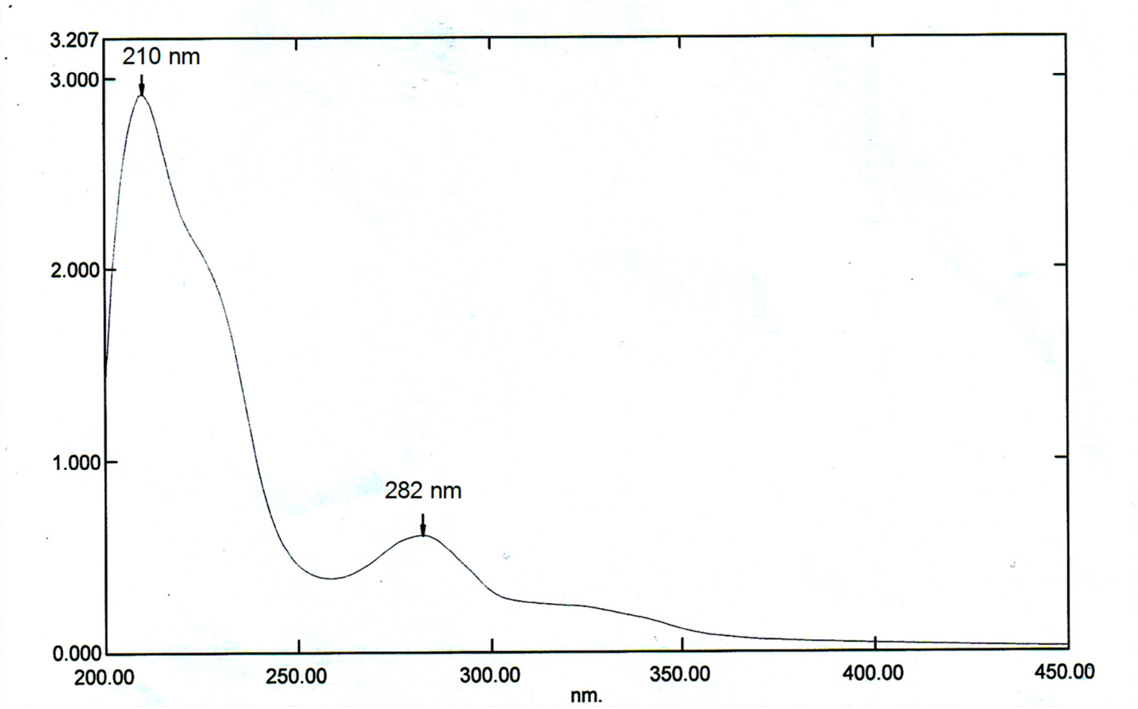

Figure 9. IR spectrum of compound **1**

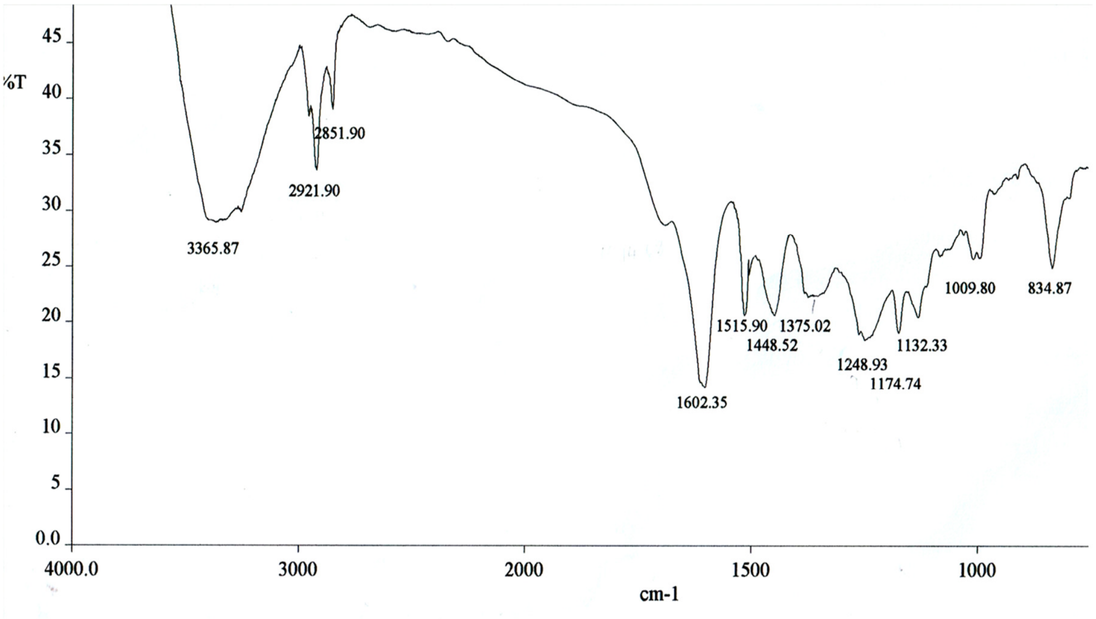

Figure 10. ^{1}H NMR spectrum of compound **2**.

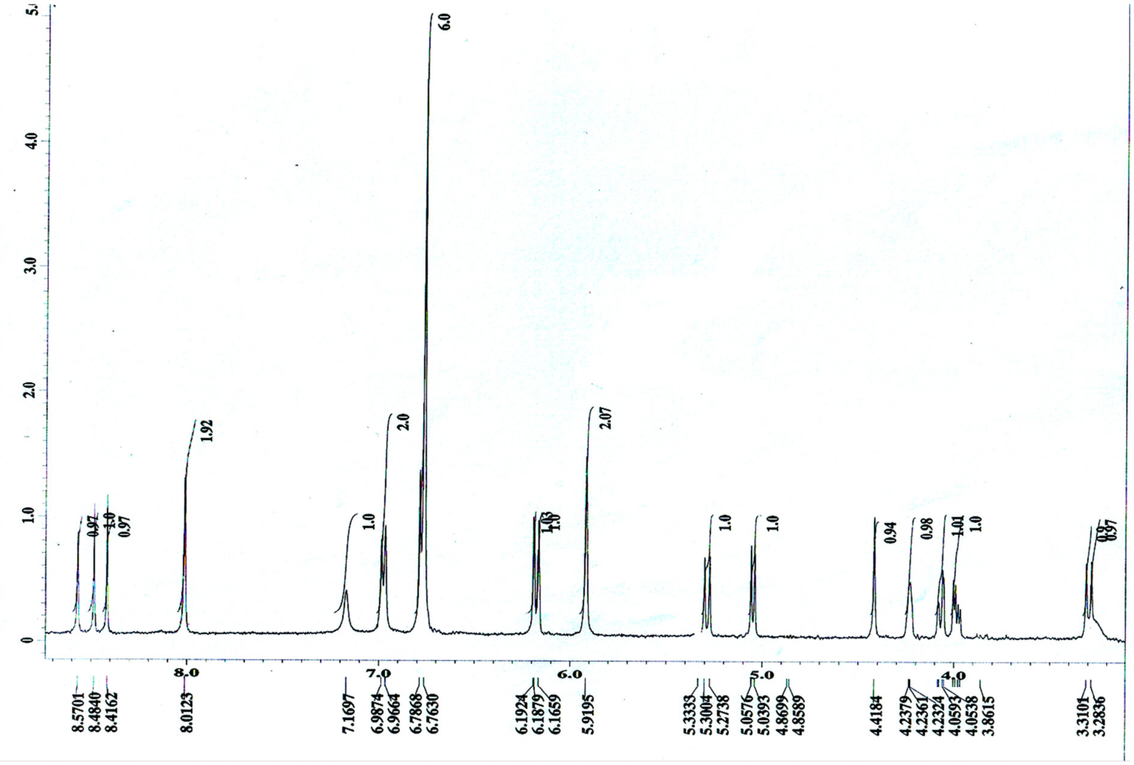

Figure 11. ^{13}C-APT NMR spectrum of compound **2**.

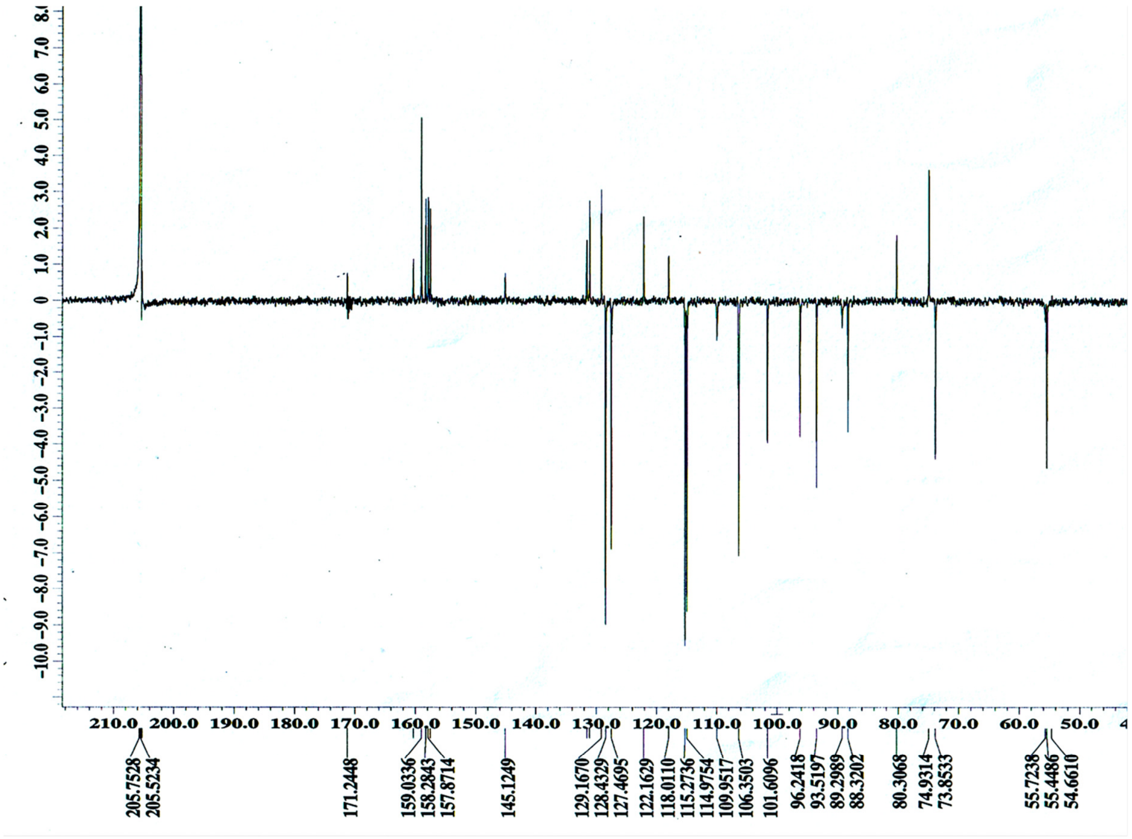

Table 1. ^{13}C-NMR and ^{1}H-NMR (100 MHz and 400 MHz,acetone-d_6) spectral data of **2**.

Position	δ_C	δ_H
1a	131.1	-
2a/6a	128.3	6.78 (m)
3a/5a	115.3	6.78 (m)
4a	157.5	-
7a	93.6	5.05 (d, 7.7)
8a	55.4	3.30 (d, 10.6)
9a	145.1	-
10a/14a	106.3	5.92 (d, 2.9)
11a/13a	158.9	-
12a	101.7	6.17 (t, 1.08,1.84)
1b	129.1	-
2b/6b	127.4	6.98 (d, 8.4)
3b/5b	114.9	6.78 (m)
4b	158.2	-
7b	89.2	5.29 (d, 10.6)
8b	55.4	3.30 (d, 10.6)
9b	131.6	-
10b	124.6	-
11b	160.3	-
12b	96.3	6.19 (d, 2.2
13b	157.8	-
14b	110.0	7.13 (br s)
1'	172.6	-
2'	80.3	-
3'	122.1	-
4'	88.3	4.44 (br s)
5'	73.9	4.24 (m)
6'	74.9	3.99 (dd, 10.6,4.8),<br>4.07 (dd, 9.2, 1.8)

Figure 12. ^{1}H-NMR spectrum of compound **3**.

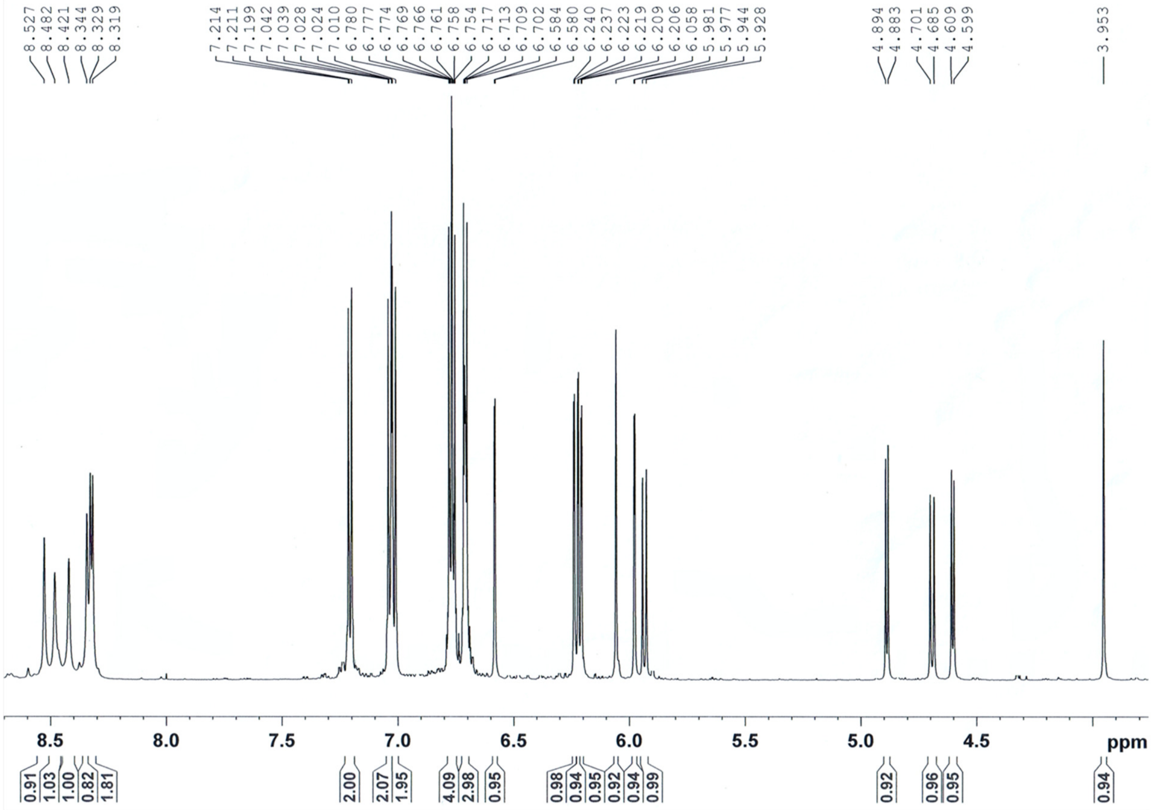

Figure 13. ^{13}C-APT NMR spectrum of compound **3**.

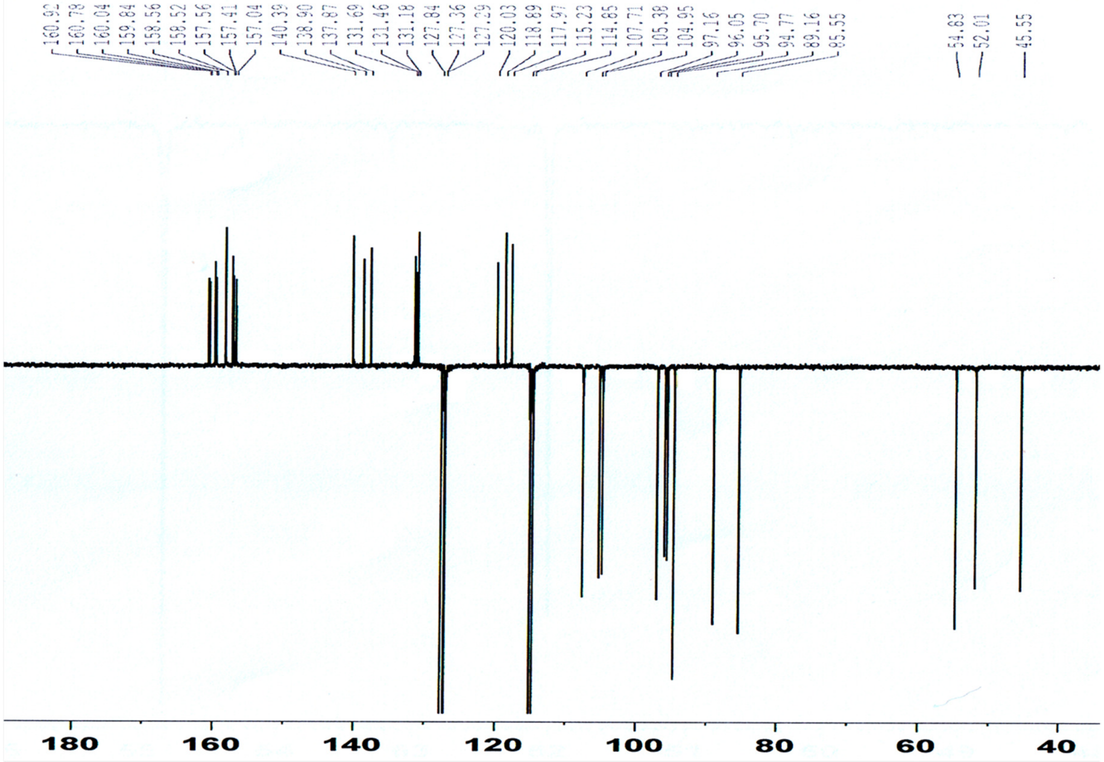

Table 2. ^{13}C-NMR and ^{1}H-NMR (150 MHz and 600 MHz, acetone-d_6) spectral data of **3**.

Position	δ_C	δ_H
1a	131.2	
2a/6a	127.3	7.21 (d, 9.0)
3a/5a	115.2	6.77 (d, 8.4)
4a	157.0	-
7a	89.2	5.94 (d, 9.6)
8a	52.0	4.69 (d, 9.6)
9a	137.8	-
10a	118.9	-
11a	159.8	-
12a	95.7	6.24 (d, 1.8)
13a	160.9	-
14a	104.9	6.58 (d, 2.4)
1b	131.7	-
2b/6b	127.8	7.04 (d, 8.4)
3b/5b	115.2	6.76 (d, 9.0)
4b	157.6	-
7b	94.8	4.89 (d, 6.6)
8b	54.8	4.60 (d, 6.0)
9b	138.9	-
10b	118.0	-
11b	158.5	-
12b	96.0	6.21 (d, 1.8)
13b	160.0	-
14b	107.7	5.98 (d, 2.4)
1c	131.5	
2c/6c	127.4	7.02 (d, 8.4
3c/5c	114.8	6.71 (d, 9.0)
4c	157.4	-
7c	85.5	6.06 (s)
8c	45.6	3.95 (s)
9c	140.4	-
10c	120.0	-
11c	158.6	-
12c	97.2	6.22 (d, 2.4)
13c	160.8	-
14c	105.4	6.71 (d, 2.4)
4a,4b,4c,13a,13b,13c (OH)		8.32, 8.33, 8.34, 8.42, 8.48, 8.53 (s)

Figure 14. ^{1}H-NMR spectrum of compound **4**.

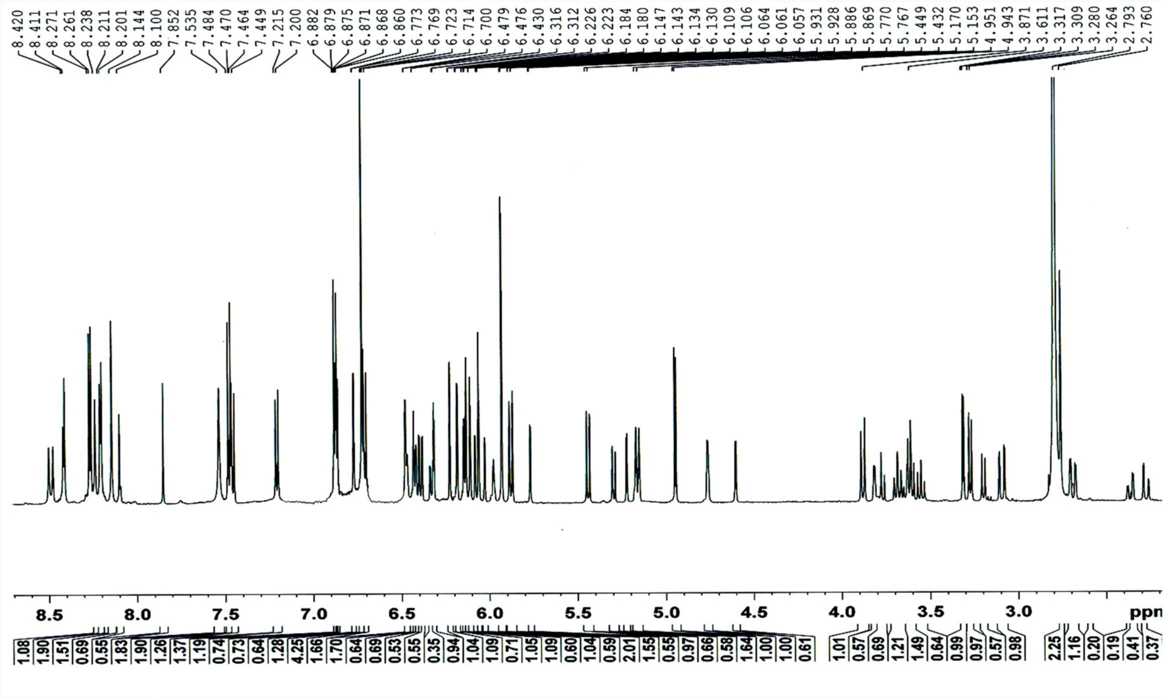

Figure 15. ^{13}C-APT NMR spectrum of compound **4**.

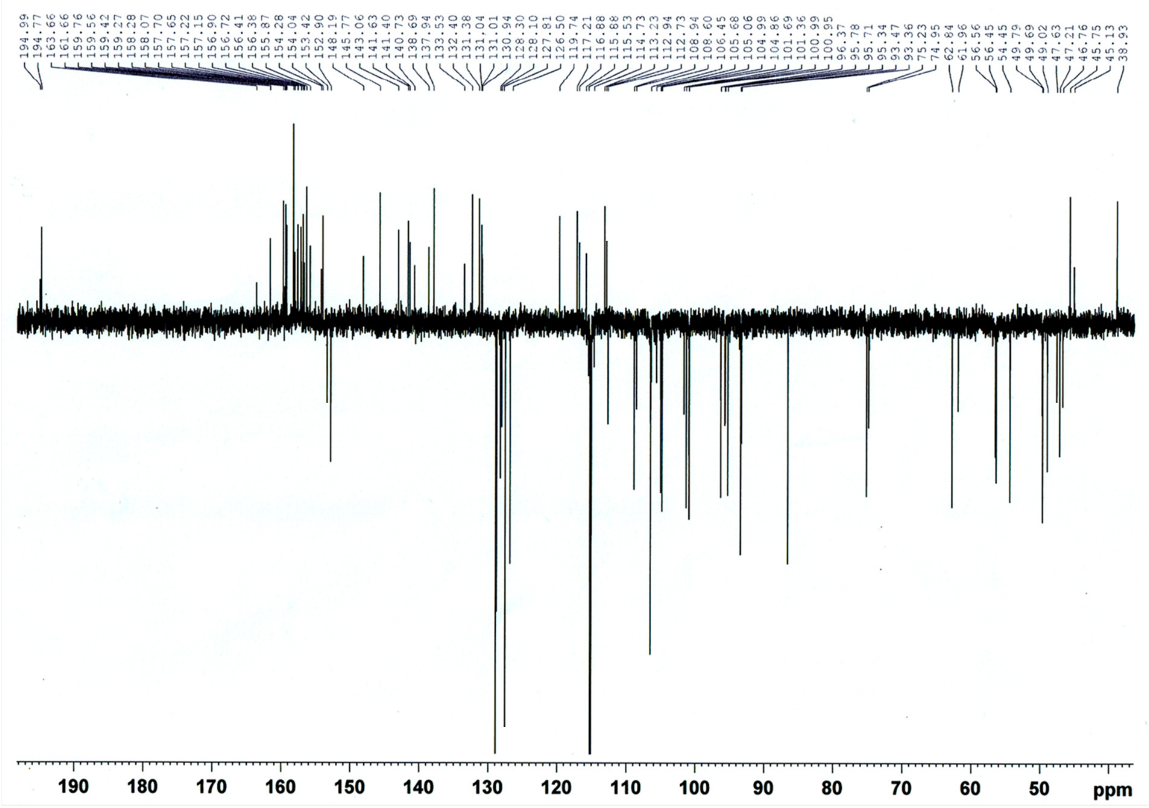

Table 3. ^{13}C-NMR and ^{1}H-NMR (150 MHz and 600 MHz, acetone-d_6) spectral data of **4**.

**Position**	**4a**		**4b**	
	**δ_C**	**δ_H**	**δ_C**	**δ_H**
1a	131.04	-	130.94	-
2a, 6a	128.96	7.48 (d, 8.6)	128.83	7.46 (d, 4.6)
3a, 5a	115.30	6.88 (d, 8.4)	115.30	6.87 (d, 9.0)
4a	157.65	-	157.70	-
7a	86.63	5.88 (d, 10.2)	86.63	5.88 (d, 10.2)
8a	49.69	5.16 (br d, 10.2)	49.79	5.16 (br d, 10.2)
9a	141.63	-	141.40	-
10a	113.23	-	112.94	-
11a	154.04	-	154.28	-
12a	100.95	6.18 (d, 2.4)	100.99	6.08 (d, 2.4)
13a	156.90	-	156.72	-
14a	105.06	6.48 (d, 1.8)	104.99	6.42 (br, s)
1b	45.75	-	45.13	-
2b	75.23	4.77 (m)	74.95	3.81 (m)
3b	38.93	2.69 (dd, 16.8, 3.0)<br>3.09 (dd, 17.4, 1.8)	38.93	2.36 (dd, 17.4, 3.6)<br>2.27 (dd, 17.4, 1.8)
4b	194.77	-	194.99	-
5b	128.30	5.44 (d, 10.2)	128.10	5.29 (d, 10.2)
6b	152.90	6.39 (dd, 10.2, 2.4)	153.42	6.33 (dd, 10.2, 2.4)
7b	47.21	3.88 (d, 12.0)	47.63	3.73 (d, 11.4)
8b	49.02	3.68 (t, 11.4, 12.0)	46.76	3.61 (t, 9.0, 11.4)
9b	137.94	-	138.69	-
10b	117.21	-	116.88	-
11b	159.42	-	159.56	-
12b	96.37	6.13 (d, 2.4)	95.78	6.14 (d, 2.4)
13b	156.38	-	157.65	-
14b	108.94	6.11 (d, 1.8)	108.60	6.02 (d, 1.8)
1c	131.38	-	131.01	-
2c/6c	NI	NI	NI	NI
3c/5c	NI	NI	NI	NI
4c	156.41	-	155.87	-
7c	62.84	3.27 (d, 9.6)	56.56	3.19 (d, 10.8)
8c	56.45	3.61 (t, 9.0, 11.4)	61.96	3.55 (t, 11.4)
9c	143.06	-	140.73	-
10c	119.74	-	115.88	-
11c	161.66	-	163.66	-
12c	95.34	6.22 (d, 1.8)	95.71	6.31 (d, 2.4)
13c	159.27	-	158.07	-
14c	104.86	6.77 (d, 2.4)	112.73	5.77 (d, 2.4)
1d	132.40	-	133.53	-
2d, 6d	127.57	6.72 (s)	126.88	7.21 (d, 9.0)
3d, 5d	115.12	6.72 (s)	115.30	6.71 (d, 8.4)
4d	157.22/157.15	-	157.15	-
7d	93.47	4.95 (d, 4.8)	93.36	5.22 (d, 2.4)

Table 3. *Cont.*

Position	4a		4b	
	δC	δH	δC	δH
8d	54.45	3.31 (d, 4.8)	56.56	4.60 (d, 2.4)
9d	145.77	-	148.19	-
10d	106.55	5.93 (d, 1.8)	105.68	5.98 (br s)
11d	158.28	-	159.76	-
12d	101.36	6.06 (t, 1.8, 2.4)	101.69	6.43 (t, 1.8, 2.4)
13d	158.28	-	159.76	-
14d	106.55	5.93 (d, 1.8)	106.45	6.47 (br, s)

4^a, 4^b, represent for major and minor conformer; NI: not identified.

Figure 16. ^{1}H-NMR spectrum of compound **5**.

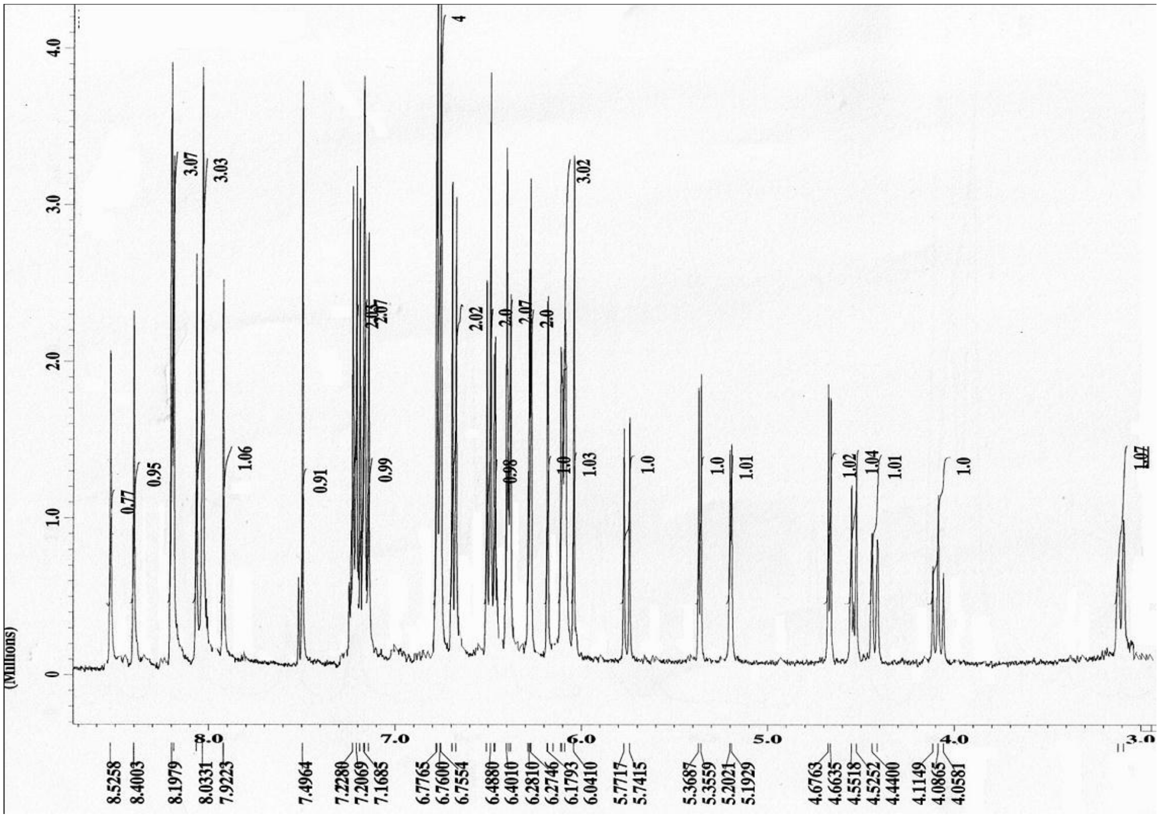

Figure 17. ^{13}C-APT NMR spectrum of compound **5**.

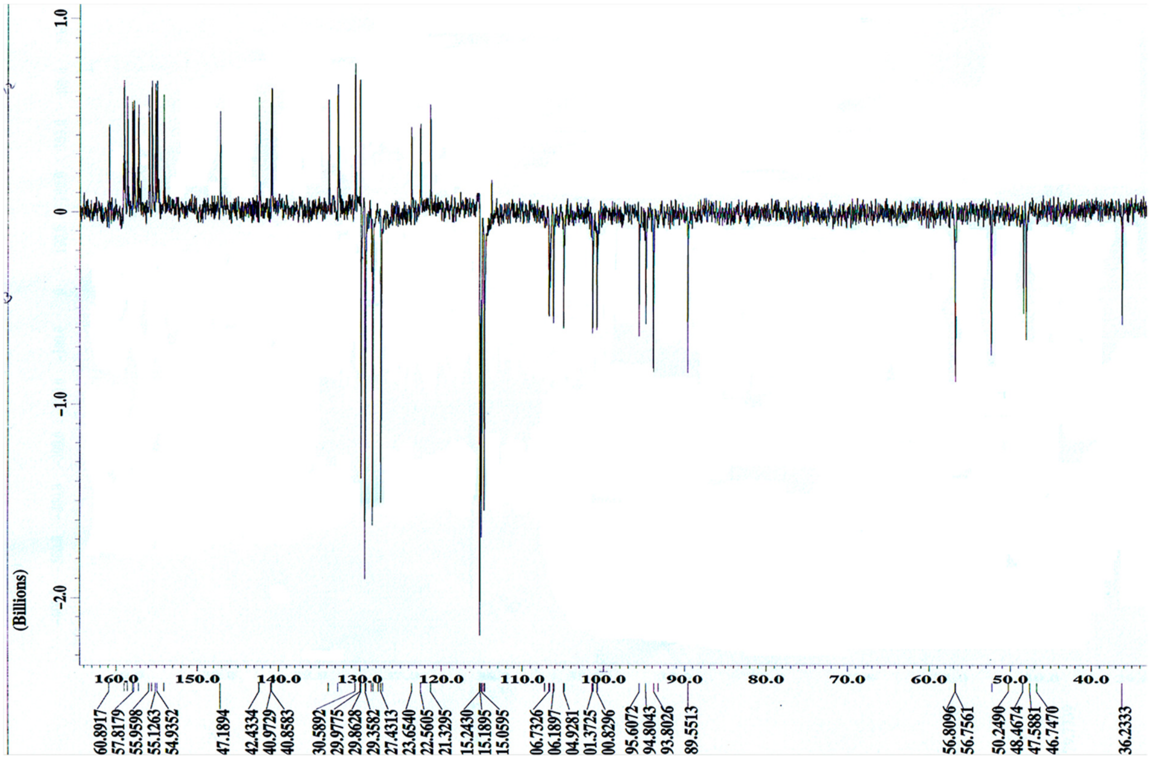

Table 4. ^{13}C-NMR and ^{1}H-NMR (100 MHz and 400 MHz, acetone-d_6) spectral data of **5**.

Position	δ_C	δ_H
1a	130.0	-
2a/6a	129.4	7.21 (d, 8.4)
3a/5a	115.2	6.77 (d, 8.4)
4a	157.8	-
7a	89.6	5.76 (d, 12.1)
8a	48.1	4.43 (d, 11.0)
9a	141.0	-
10a	123.7	-
11a	154.9	-
12a	100.8	6.28 (d, 2.2)
13a	156.0	-
14a	104.9	6.11(d, 2.6)
1b	132.7	-
2b/6b	130.0	7.15 (d, 8.4)
3b/5b	114.7	6.69 (d, 8.4)
4b	155.1	-
7b	36.2	5.20 (d, 3.7)
8b	52.3	3.11 (br d, 11.3)
9b	142.4	-
10b	114.9	-
11b	158.0	-

Table 4. ***Cont.***

Position	δ_C	δ_H
12b	95.6	6.04 (s)
13b	154.1	-
14b	121.3	-
1c	130.6	-
2c/6c	128.4	6.39 (d, 8.4)
3c/5c	115.1	6.50 (d, 8.4)
4c	155.6	-
7c	56.8	4.09 (t, 11.4)
8c	48.5	4.54 (d, 10.6)
9c	140.9	-
10c	122.6	-
11c	160.9	-
12c	94.8	6.18 (d, 2.2)
13c	158.6	-
14c	106.2	6.47 (d, 2.2)
1d	133.9	-
2d/6d	127.4	7.18 (d, 8.8)
3d/5d	115.1	6.77 (d, 8.4)
4d	157.2	-
7d	93.8	5.36 (d, 5.1)
8d	56.8	4.67 (d, 5.1)
9d	147.2	-
10d/14d	106.7	6.09 (br s)
11d/13d	159.0	-
12d	101.4	6.27 (t, 2.2)

Figure 18. ^{1}H NMR spectrum of compound **6**.

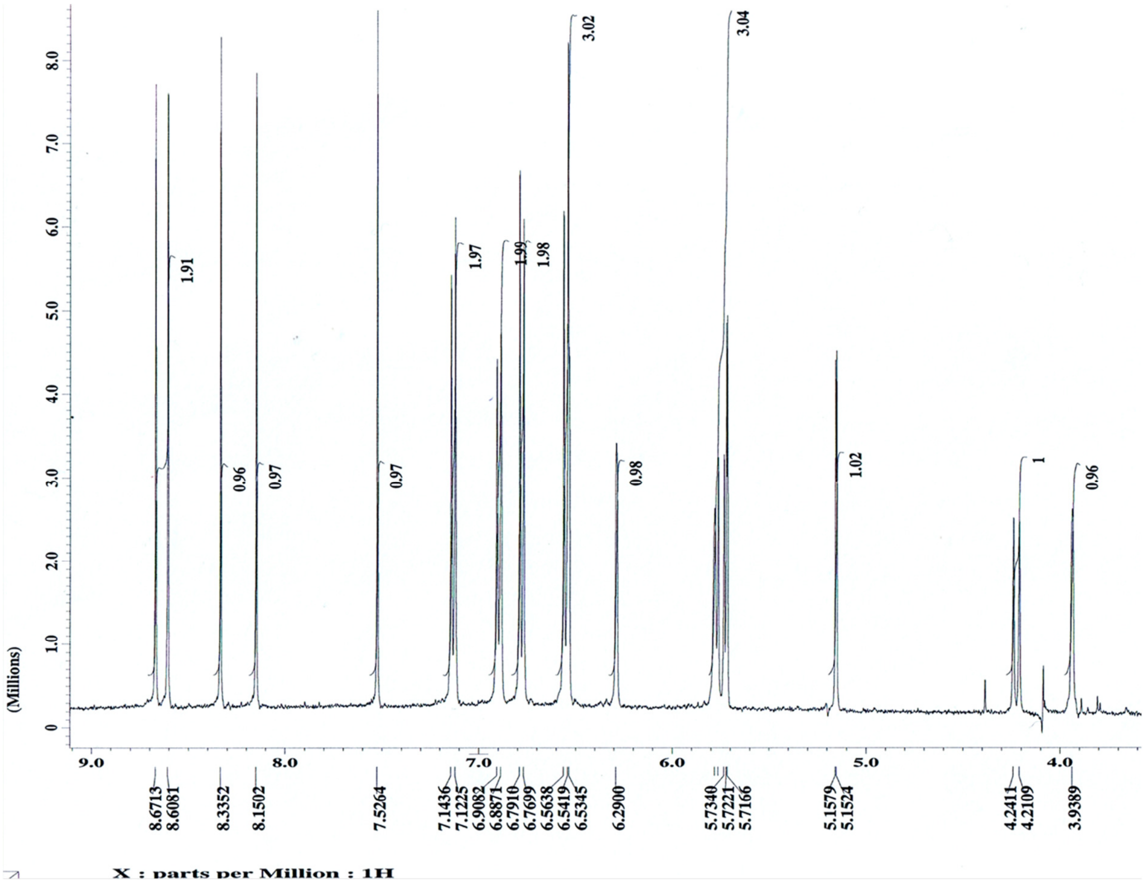

Figure 19. ^{13}C-APT NMR spectrum of compound **6**.

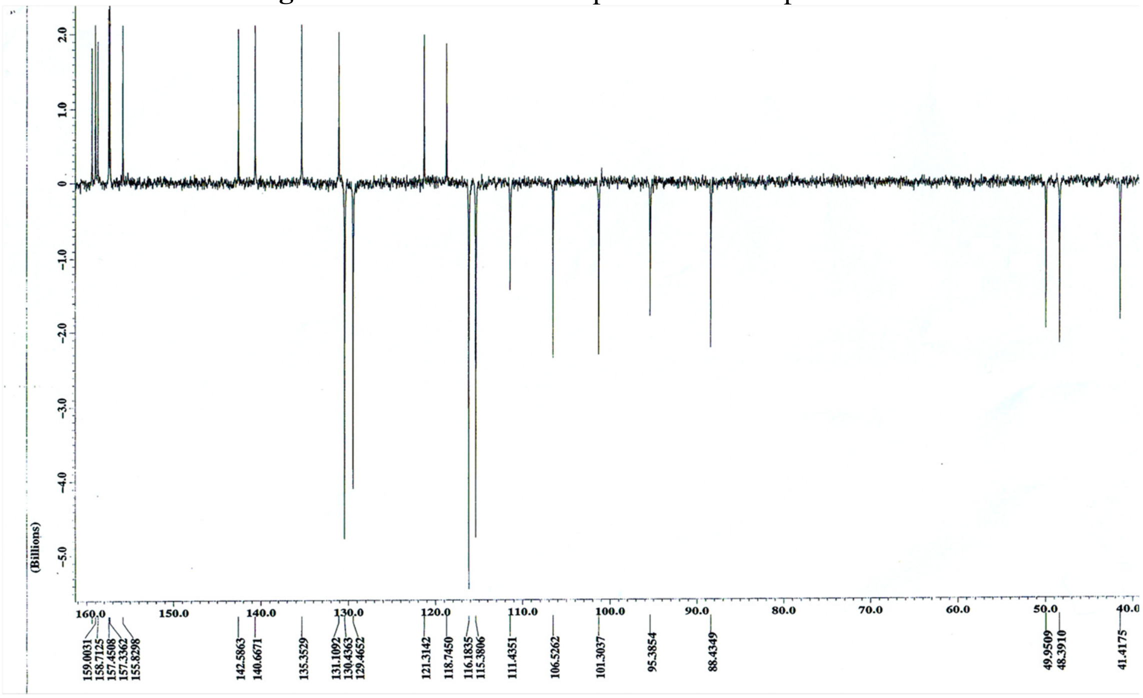

Table 5. ^{13}C-NMR and ^{1}H-NMR (100 MHz and 400 MHz, acetone-d_6) spectral data of **6**.

Position	δ_C	δ_H
1a	131.1	-
2a/6a	130.4	7.12 (d, 8.4)
3a/5a	115.4	6.77 (d, 8.4)
4a	158.7	-
7a	88.4	5.74 (d, 12.1)
8a	49.9	4.22 (d, 12.1)
9a	142.6	-
10a	121.3	-
11a	159.0	-
12a	101.3	6.53 (br s)
13a	157.4	-
14a	106.5	6.28 (br s)
1b	135.4	-
2b/6b	129.5	6.89 (d, 8.1)
3b/5b	116.2	6.54 (d, 8.8)
4b	155.8	-
7b	41.4	5.77 (br s)
8b	48.4	3.93 (s)
9b	140.7	-
10b	118.7	-
11b	159.5	-
12b	95.4	5.71 (d, 2.2)
13b	157.3	-
14b	111.4	5.15(d, 2.2)
